# Prediction of Biochemical Recurrence Based on Molecular Detection of Lymph Node Metastasis After Radical Prostatectomy

**DOI:** 10.1016/j.euros.2022.07.005

**Published:** 2022-08-16

**Authors:** Berna C. Özdemir, Nicolas Arnold, Achim Fleischmann, Janine Hensel, Irena Klima, Marianna Kruithof-de Julio, Fiona Burkhard, Stefanie Hayoz, Bernhard Kiss, George N. Thalmann

**Affiliations:** a Urological Research Laboratory and Department of Urology, University of Bern, Inselspital, Bern, Switzerland; b Department of Oncology, University of Bern, Inselspital, Bern, Switzerland; c Institute of Pathology, University of Bern, Bern, Switzerland; d Swiss Group for Clinical Cancer Research (SAKK) Coordinating Center, Bern, Switzerland

**Keywords:** Prostate cancer, Lymph nodes, Metastasis, Extended lymphadenectomy, Micrometastasis, Isolated tumor cells, Molecular detection, Molecular markers, mRNA expression

## Abstract

**Background:**

Molecular detection of lymph node (LN) micrometastases by analyzing mRNA expression of epithelial markers in prostate cancer (PC) patients provides higher sensitivity than histopathological examination.

**Objective:**

To investigate which type of marker to use and whether molecular detection of micrometastases in LNs was predictive of biochemical recurrence.

**Design, setting, and participants:**

LN samples from PC patients undergoing radical prostatectomy with extended LN dissection between 2009 and 2011 were examined for the presence of micrometastases by both routine histopathology and molecular analyses.

**Outcome measurements and statistical analysis:**

The mRNA expression of a panel of markers of prostate epithelial cells, prostate stem cell–like cells, epithelial-to-mesenchymal transition, and stromal activation, was performed by quantitative real-time polymerase chain reaction. The expression levels of these markers in LN metastases from three PC patients were compared with the expression levels in LN from five control patients without PC in order to identify the panel of markers best suited for the molecular detection of LN metastases. The predictive value of the molecular detection of micrometastases for biochemical recurrence was assessed after a follow-up of 10 yr.

**Results and limitations:**

Prostate epithelial markers are better suited for the detection of occult LN metastases than molecular markers of stemness, epithelial-to-mesenchymal transition, or reactive stroma. An analysis of 1023 LNs from 60 PC patients for the expression of prostate epithelial cell markers has revealed different expression levels and patterns between patients and between LNs of the same patient. The positive predictive value of molecular detection of occult LN metastasis for biochemical recurrence is 66.7% and the negative predictive value is 62.5%. Limitations are sample size and the hypothesis-driven selection of markers.

**Conclusions:**

Molecular detection of epithelial cell markers increases the number of positive LNs and predicts tumor recurrence already at surgery.

**Patient summary:**

We show that a panel of epithelial prostate markers rather than single genes is preferred for the molecular detection of lymph node micrometastases not visible at histopathological examination.

## Take Home Message

Our study shows that molecular detection of lymph node micrometastases with a panel of prostate epithelial markers seems to be more useful for early detection of biochemical recurrence than histology alone or using markers of stem cells, epithelial-to-mesenchymal transition, or reactive stroma.

## 1 Introduction

Organ-confined prostate cancer (PC) is cured by radical prostatectomy (RP) in only 70–80% of patients. This is mainly due to early dissemination of cancer cells and formation of occult metastases not manifest at the time of treatment of the primary tumor [1]. It is currently difficult to unequivocally identify high-risk patients in need of close, long-term follow-up.

The presence of histologically detectable pelvic lymph node (LN) metastases (pN1) is an important predictor of disease recurrence [2]. However, over 20% of patients classified as LN negative (pN0) will suffer recurrence despite effective local therapy [3]. This suggests that a conventional histopathological analysis of pelvic LNs may miss small metastatic foci. The discovery rate of micrometastases can be increased by immunohistochemical staining with antibodies against cytokeratins and prostate-specific antigen (PSA) [4], [5]. Additional sensitivity is attained by quantitative real-time polymerase chain reaction (RT-qPCR) measuring the expression of PSA and prostate-specific membrane antigen (PSMA) [6], [7], [8]. A molecular LN analysis can identify pN0 patients with a higher risk of biochemical recurrence [6], [7], [8] and is superior to histopathological LN status [9]. The outcome of patients with occult LN metastases detected by molecular analyses is similar to that of pN1 patients [10].

Most studies on LN micrometastasis in PC patients have focused on the detection of prostate epithelial markers such as PSA and PSMA [6], [7], [8]. However, other markers may be more helpful in detecting LN micrometastases. In fact, PC circulating tumor cells (CTCs) present in peripheral blood [11] and early disseminating cancer cells found in bone marrow have a stem cell (SC) phenotype [12] and undergo epithelial-to-mesenchymal transition (EMT) [13], and may therefore escape detection based on epithelial markers. Metastasis-initiating cells most likely represent a subpopulation of CTCs and, consequently, probably also express SC features [14]. On the contrary, the presence of a reactive stroma in the primary tumor as well as alteration of the LN microenvironment was shown to predict recurrence-free survival after RP [15]. There are currently only limited data on the ideal number and type of markers for the molecular detection of PC recurrence.

In this prospective study, we assessed the expression of a panel of molecular markers in LNs from patients with organ-confined PC who were followed up for 10 yr. We investigated whether the additional assessment of mRNA expression of markers of prostate SC-like, EMT, and reactive stroma determined in our laboratory could increase the detection rate of LN micrometastases in PC patients. Finally, we evaluated the predictive value of molecular detection of LN metastases for biochemical recurrence.

## 2 Patients and methods

### 2.1 Surgical specimens

Tissue sampling was approved by the local ethical committee (number 06/03). Between March 2009 and August 2011, LN specimens were obtained from 60 PC patients with cT1–4 cN0 cM0 disease (Union for International Cancer Control 2009 edition) undergoing RP and extended lymphadenectomy at the Department of Urology, University of Bern.

LNs were meticulously searched for during the pathological examination and counted according to their specific location and side. Then each identified LN was cut in half, one half was stored in RNAlater for RNA extraction and the other half to be used for histopathological examination was fixed in formalin, and the fatty tissue of lymphadenectomy specimens was dissolved in acetone after formalin fixation. The cut surface of each LN half was examined by eye, and if macroscopically metastases were suspected, then this half was used for histopathological assessment and the other half was used for molecular analysis. All LN halves were embedded in paraffin. Each tissue block was cut into 5 mm sections and stained with hematoxylin-eosin. One section per block was microscopically analyzed for metastases by the pathologist. The length and width of the metastatic deposits were measured. If necessary, an immunohistochemical analysis was carried out.

### 2.2 Gene expression analysis

RNA extraction, cDNA synthesis, and RT-qPCR were performed as previously described [16]. Gene symbols and corresponding expression assays are listed in Supplementary Table 1.

Control LN specimens were obtained from four female patients undergoing surgery for noncancerous reasons and from one patient undergoing Millin’s prostatectomy.

### 2.3 Immunohistochemistry

Immunohistochemical staining was performed on 47 deparaffinized LN sections of six pN1 patients with the primary antibodies listed in Supplementary Table 2, as previously described [17].

### 2.4 Endpoints and follow-up

Follow-up information of the PSA level and further PC treatment was updated regularly, the last time being January 10, 2022. Adjuvant radiotherapy and/or androgen deprivation therapy after RP was administered based on histopathology results and defined as postoperative treatment without evidence of biochemical recurrence. After reaching an undetectable value, a confirmed postoperative PSA value of >0.2 µg/l as well as a detectable PSA value of ≥0.1 µg/l 3 mo postoperatively (PSA persistence) was considered biochemical recurrence. Biochemical recurrence-free survival (bRFS) was calculated from the date of surgery until biochemical recurrence. Patients with no biochemical recurrence were censored at the last follow-up date.

### 2.5 Statistical analysis

Statistical analysis was performed with GraphPad Prism version 6.0d (www.graphpad.com) and R version 4.0.3. (www.r-project.org). The Mann-Whitney test was used to compare mRNA expression between LNs from PC and control patients.

The Kruskal-Wallis test was applied to assess the association of LN status with clinical variables. The Spearman correlation coefficient rho was used to assess the presence and magnitude of monotonous trends between the level of evidence of LN metastases (pN0/molN0 < pN0/molN1 < pN1/molN1) and ordinal clinical risk factors. The median bRFS as well as bRFS at 10 yr together with 95% confidence intervals (CIs) was calculated using the Kaplan-Meier method and compared between LN status using the log-rank test. Hazard ratios (HRs) and 95% CIs were calculated using Cox regression models. All statistical tests performed were two sided, and a *p* value of <0.05 was considered statistically significant.

## 3 Results

### 3.1 Expression of candidate marker genes in LNs of control and PC patients

We have compared the expression levels of candidate genes in seven LNs from three patients with macroscopic PC LN metastases and in 11 LNs from five control patients.

**Fig. 1:**
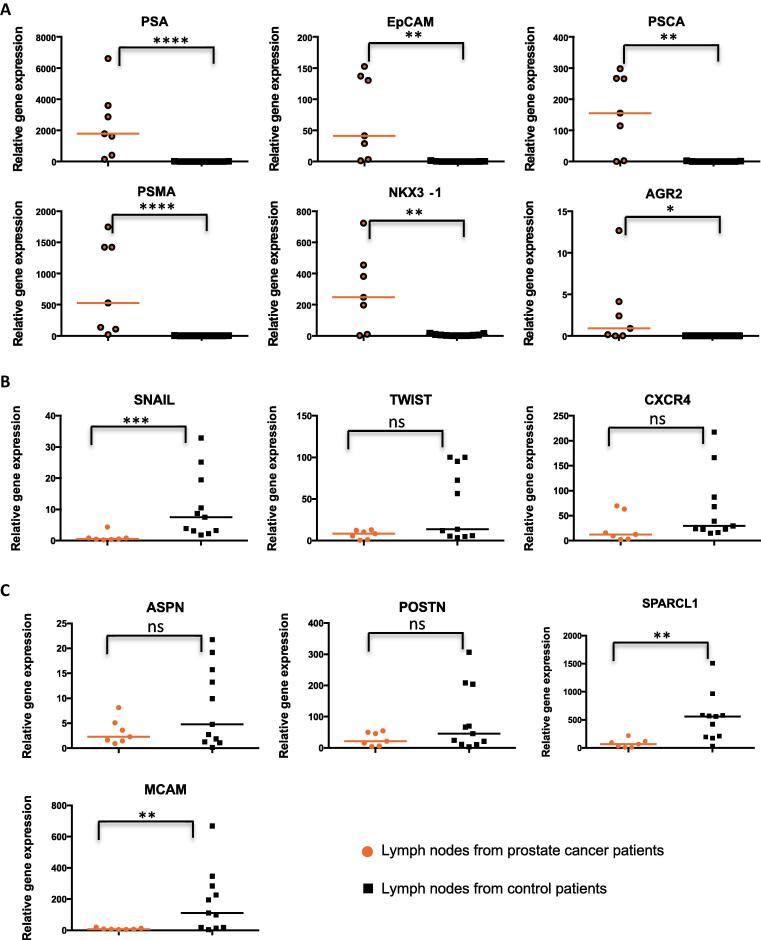
The mRNA expression levels of (A) prostate epithelial cell markers, (B) epithelial–to-mesenchymal transition (EMT) markers, and (C) reactive stroma markers in lymph nodes of prostate cancer (orange) compared with control patients (black). PSA = prostate-specific antigen; PSCA = prostate stem cell antigen; PSMA = prostate-specific membrane antigen. *p < 0.05. **p < 0.001. ***p < 0.0005. ****p < 0.0001. ns = *p* > 0.05.

All markers of epithelial cells (*PSA*, *EpCAM*, *PSCA*, *PSMA*, *NKX3-1*, and *AGR2*) were expressed at a significantly higher level in LNs from PC patients than in those from control patients (Fig. 1A). In contrast, the levels of expression of the EMT markers (*SNAIL*, *TWIST*, and *CXCR4*) were either higher in control patients or not different between the two groups of patients (Fig. 1B). The results were similar for the markers of reactive stroma (*ASPN*, *POSTN*, *SPARCL1*, and *MCAM*; Fig. 1C).

**Fig. 2:**
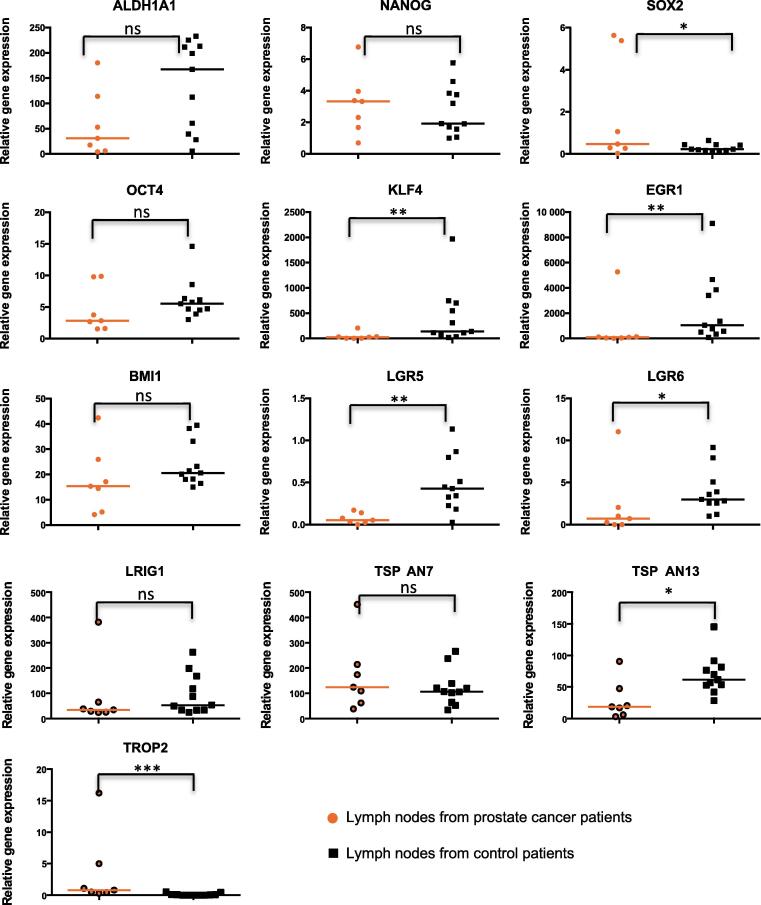
The mRNA expression levels of stem cell markers in lymph nodes of prostate cancer (orange) compared with control patients (black). *p < 0.05. **p < 0.001. ***p < 0.0005. ns = *p* > 0.05.

With the exception of *TROP2*, all proven or putative markers of SCs (*ALDH1A1*, *NANOG*, *SOX2*, *OCT4*, *KLF4*, *EGR1*, *BMI1*, *LGR5*, *LGR6*, *LRIG1*, *TSPAN7*, and *TSPAN13)* were expressed in LNs from control patients to similar or even higher levels than in PC patients (Fig. 2). *TROP2* expression was barely detectable in LNs from controls but could be measured in a few LNs from a PC patient.

Based on these results, we have selected the panel of epithelial markers to be further assessed for molecular detection of micrometastases in our cohort of 60 patients.

### 3.2 Immunohistochemical detection of prostate epithelial markers in LNs from PC patients

**Fig. 3:**
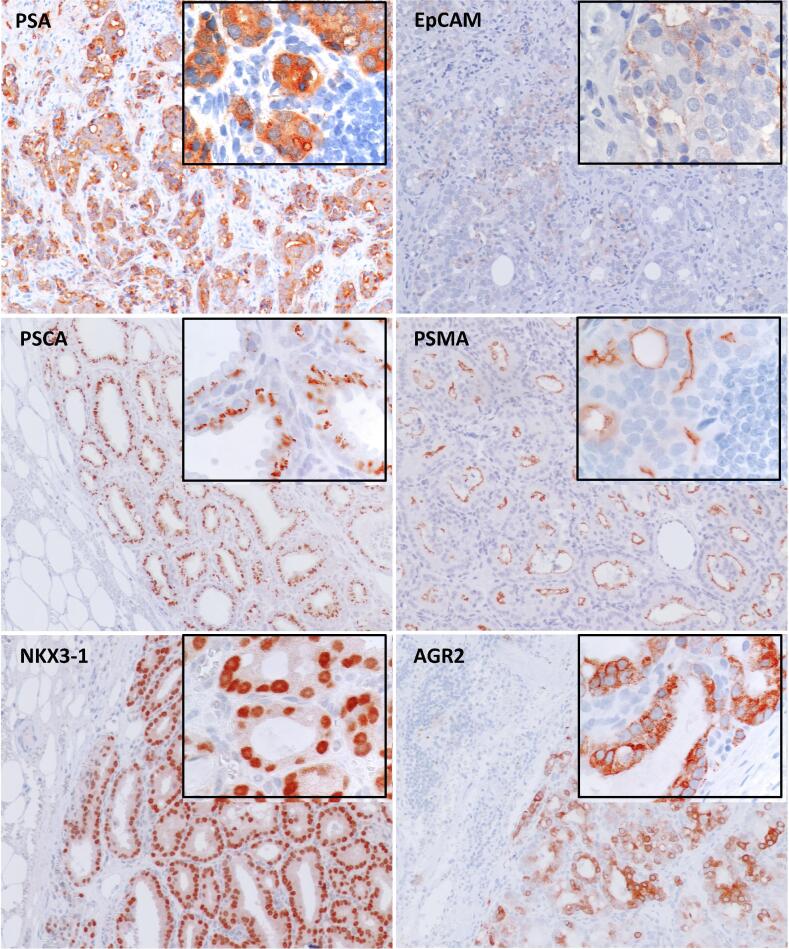
Protein expression of lymph node section from prostate cancer patients with macroscopic metastases (pN1) at 10× and 60× magnification (insert). PSA = prostate-specific antigen; PSCA = prostate stem cell antigen; PSMA = prostate-specific membrane antigen.

Protein expression of PSA, PSCA, EpCAM, PSMA, NKX3-1, and AGR2 was analyzed on LN sections from six pN1 patients. Tumor cells in LNs showed cytoplasmic expression of PSA, PSCA, and AGR2; membrane and cytoplasmic expression of PSMA and EpCAM; and nuclear expression of NKX3-1. Lymphoid cells were negative for all markers (Fig. 3).

### 3.3 PC patient and LN characteristics

The clinical and pathological characteristics of the patients are summarized in Table 1. The patients were followed up until January 10, 2022 (median follow-up, 11.3 yr; range, 1.3–12.8 yr). Seven patients (12%) died and five patients (8.3%) were lost to follow-up.

**Table 1:** Clinical and pathological characteristics of patients

	Total	pN0	pN1
No. of patients	60	48	12
Mean age at time of surgery (yr) Gleason score	62.7	62.6	63.0
≤6	8	8	0
7 (3 + 4)	25	23	2
7 (4 + 3)	5	12	3
8 and 9	12	5	7
Clinical stage			
T2a	5	4	1
T2b	0	0	0
T2c	32	31	1
T3a	7	6	1
T3b	15	7	8
T4	1	0	1
No. of patients with biochemical recurrence	32	21	12
Median time to relapse (mo)	26	50	3

A total of 2108 LNs (median per patient, 33.5; range, 13–74) obtained from 60 PC patients were screened by histopathology. Out of them, 63 LNs from 12 patients (20%) showed histological evidence of metastases (pN1). A total of 1023 LNs (median per patient, 17; range, 5–34) from the same 60 patients were screened for mRNA expression of *PSA*, *PSCA*, *EpCAM*, *PSMA*, and *NKX3-1*. Various levels of expression of these genes were measured. The threshold for positive expression of these markers was set based on the mean expression levels + 2SD measured in 11 LNs of five control patients (*PSA*: 16.23, *PSCA*: 1.99, *EpCAM*: 0.57, *PSMA*: 1.39, and *NKX3-1*: 9.27; Supplementary Table 3).

Positive *PSA* mRNA expression was measured in LNs of eight pN1 patients (67%), but also in LNs from three pN0 patients (6.25%). *EpCAM* expression was detectable in nine pN1 patients (75%) and two of the pN0 patients (4%). *PSCA* expression could be measured in six pN1 patients (50%) but in none of the pN0 patients. *PSMA* mRNA expression was detectable in LNs from 11 pN1 (92%) and 23 pN0 patients (50%). Expression of *NKX3-1* mRNA was found in nine pN1 patients (75%), and 12 pN0 patients (25%; Table 2).

**Table 2:** Results of the histopathological and molecular analysis of the expression of prostate epithelial cell markers

	Total	pN0	pN1
No. of patients	60	48	12
Histological examination			
No. of lymph nodes analyzed	2108	1694	414
No. of lymph nodes with metastases	63	0	63
Gene expression analysis			
No. of lymph nodes analyzed	1023	827	196
No. of *PSA*^pos^ lymph nodes (patients)	61 (11)	9 (3)	52 (8)
No. of *EpCAM*^pos^ lymph nodes (patients)	62 (11)	8 (2)	53 (9)
No. of *PSCA*^pos^ lymph nodes (patients)	33 (6)	0 (0)	33 (6)
No. of *PSMA*^pos^ lymph nodes (patients)	133 (35)	57 (23)	78 (11)
No. of *NKX3-1*^pos^ lymph nodes (patients)	71 (20)	23 (12)	100 (9)

EpCAM = epithelial cell adhesion molecule; NKX3-1 = homeobox protein Nkx-3.1; PSA = prostate-specific antigen; PSCA = prostate stem cell antigen; PSMA = prostate-specific membrane antigen.

**Fig. 4:**
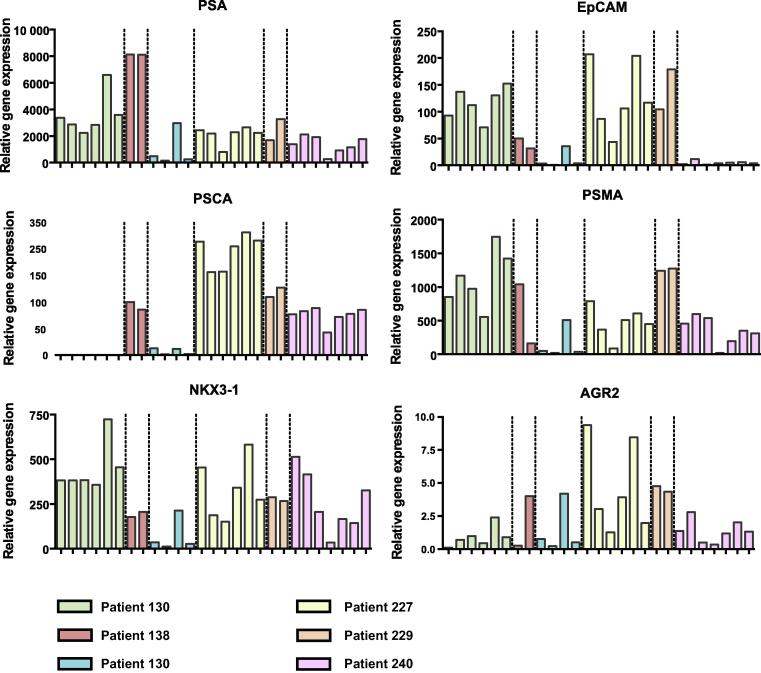
The mRNA expression pattern for *PSA*, *PSCA*, *EpCAM*, *PSMA*, *NKX3-1*, and *AGR2* among LNs of the same patient, as shown for representative LNs of six pN1 patients. LN = lymph node; PSA = prostate-specific antigen; PSCA = prostate stem cell antigen; PSMA = prostate-specific membrane antigen.

In general, the pattern of expression of *PSA*, *PSCA*, *EpCAM*, *PSMA*, *NKX3-1*, and *AGR2* was very heterogeneous among LNs of the same patient, as shown for representative LNs of six pN1 patients (Fig. 4).

### 3.4 mRNA expression of prostate epithelial markers in LNs from patients with biochemical recurrence

With a median follow-up of 11.3 yr, 33 patients experienced biochemical recurrence (55%). Of them, 12 had been staged as pN1 and 21 as pN0. The biochemical recurrence rate was therefore 100% among the 12 pN1 patients and 43.8% among the 48 pN0 patients. The median bRFS was 10 yr (95% CI [5.3–11.9]) overall, and 0.3 yr (95% CI [0.3–5.3]) and 11.8 yr (95% CI [6.4–not reached]) in pN1 and pN0 patients, respectively (log-rank *p* < 0.0001, HR 5.42 [95% CI {2.60–11.29}]).

Eleven of the 12 pN1 patients (91.7%) showed expression of one or multiple epithelial markers (pN1/molN1). Of the 21 pN0 patients who relapsed, eight (38.1%) showed no expression of any molecular marker (pN0/molN0), while 13 (61.9%) were positive for one or more marker type (pN0/molN1). Twelve patients overall were positive in the molecular analysis with no sign of recurrence. Fifteen patients were negative in the analysis and had no sign of recurrence (Table 3).

**Table 3:** Biochemical recurrence in reference to histopathological and molecular positivity

	Total	BCR–	BCR+
pN0	48	27 (56.3%)	21 (43.8%)
pN1	12	0 (0%)	12 (100%)
molN0	24	15 (62.5%)	9 (37.5%)
molN1	36	12 (33.3%)	24 (66.7%)
pN0/molN0	23	15 (65.2%)	8 (34.8%)
pN0/molN1	25	12 (48.0%)	13 (52.0%)
pN1/molN0	1	0 (0%)	1 (100%)
pN1/molN1	11	0 (0%)	11 (100%)

BCR = biochemical recurrence.

**Fig. 5:**
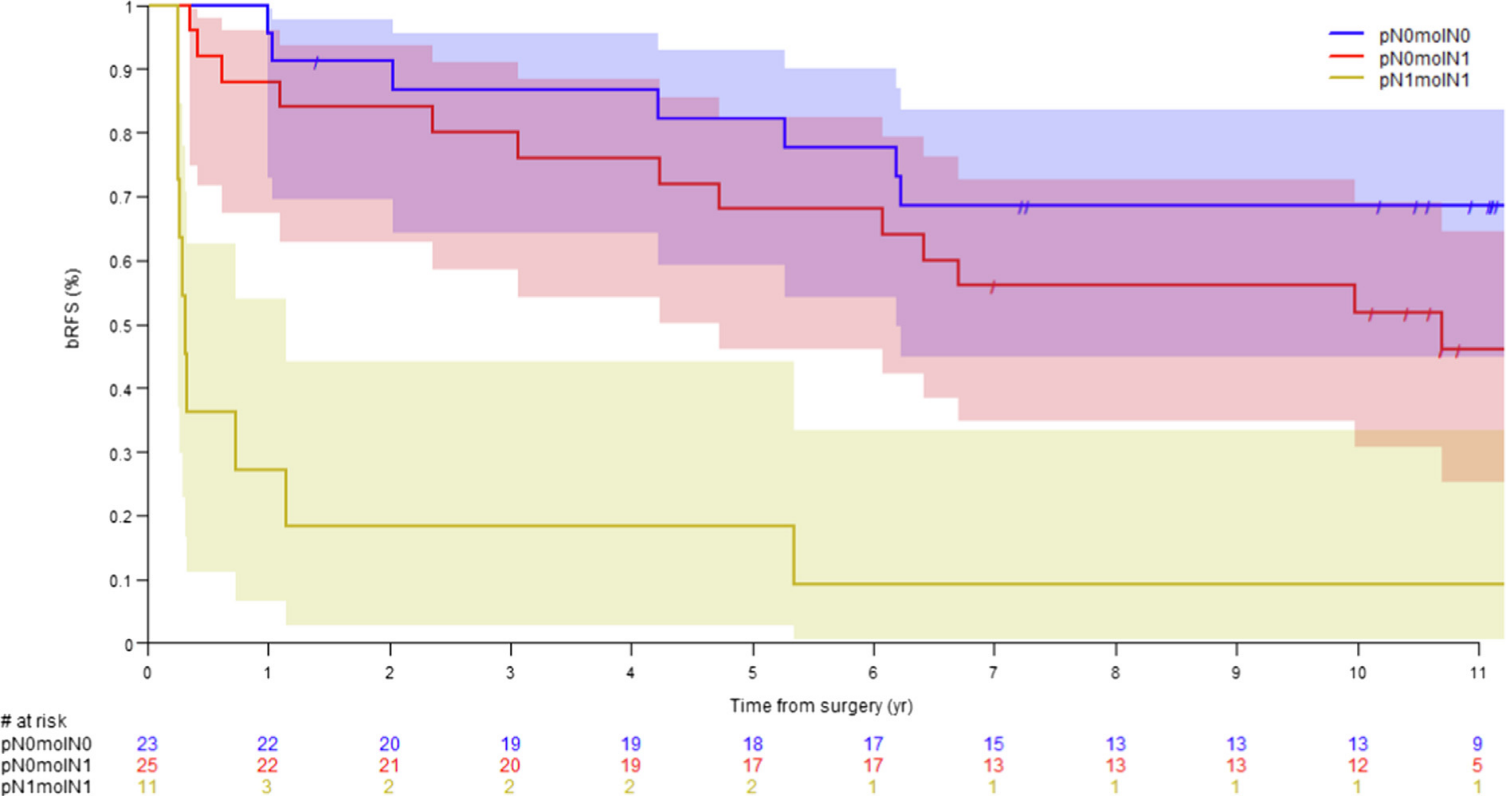
Median biochemical recurrence-free survival (bRFS) in pN0 patients with (pN0molN1) and without (pN0molN0) molecular detection of markers, and pN1 patients with molecular detection of markers (pN1molN1).

The median bRFS was 5.7 yr (95% CI [1.1–11.9]) and 11.8 yr (95% CI [6.2–not reached]) in molN1 and molN0 patients, respectively (log-rank *p* = 0.013, HR 2.57 [95% CI {1.18, 5.59}]). The median bRFS was 0.3 yr (95% CI [0.3–1.1]) for pN1/molN1 patients, 10.7 yr (95% CI [4.7–not reached]) for pN0/molN1 patients, and not reached (95% CI [6.2–not reached]) for pN0/molN0 patients. The one pN1/molN0 patient recurred after 7.8 yr (Fig. 5). The positive predictive value of molecular detection of epithelial cell markers was 66.7% (95% CI 49.0–81.4%) and the negative predictive value was 62.5% (95% CI 40.6–81.2%).

## 4 Discussion

Molecular detection of micrometastases in LNs from PC patients with localized disease has the potential to refine the diagnosis of high-risk PC patients. In this study, we investigated whether an extended panel of markers for prostate epithelial cells, SCs/progenitor cells, EMT, and reactive stroma would be useful to reveal the presence of occult metastases.

Our data confirm previous reports demonstrating that molecular screening of LNs could identify patients at risk of biochemical recurrence, although they had been classified to have pN0 by histopathology [9]. However, 38.1% of the pN0 patients not showing any molecular marker expression (pN0/molN0) experienced biochemical recurrence. This absence of molecular positivity may be due to several reasons such as local recurrence rather than nodal metastases or due to the insufficient sensitivity of our detection assay as well as possibly quenching of the positive PCR result by the LN microenvironment of otherwise prognostic markers. On the contrary, all pN1 patients except one were also positive in the molecular screening. Nevertheless, when each LN was considered, there was not always an overlap between RNA expression data and histopathological findings. This discrepancy may be due to the fact that, since only half of each LN was analyzed by one of the methods, the metastatic foci may have been located in one half only. Small metastases might also have been missed by the histopathological analysis since it was limited to one section per LN. In addition, in 33.3% of the cases with positive PCR results, the patients did not experience recurrence during the follow-up time. It is conceivable that in these cases, the molecular markers identify a population of dormant cancer cells in the LN. This state has been called “micrometastatic dormancy” by Ruppender and colleagues [18] and defines a group of tumor cells that cannot grow due to a restrictive proliferation/apoptosis equilibrium.

It is currently not clear whether the population of patients with molecular detection of LN metastases might benefit from adjuvant therapies, and further research should address this question.

Notably, our results validate epithelial markers as the best markers for the detection of LN metastases. Besides the widely used epithelial markers *PSA*, *EpCAM*, and *PSMA*, we also measured the mRNA expression levels of *PSCA*, *AGR2*, *NKX3-1*, *TROP2*, and the *TMPRSS2-ERG* fusion gene. PSCA is a protein overexpressed in the majority of PC patients, and we have previously shown that SCs/progenitor cells from the primary tumor of PC patients express PSCA [19]. High PSCA expression has been correlated with poor prognosis and progression toward neuroendocrine PC [20], and is being investigated as a target for CAR T-cell therapy for castration-resistant PC [21]. Likewise, AGR2 is overexpressed in PC and is implicated in the control of cellular senescence [22]. The homeobox gene *NKX3-1* involved in differentiation of the normal prostate epithelium is a very sensitive and specific marker of PC metastases [23]. TROP2 is a marker of prostate basal cells with SC characteristics, and high TROP2 expression by immunohistochemistry was recently shown to predict biochemical recurrence after RP [24]. Presence of TMPRSS2-ERG fusion in the LN did not prove to be a prognostic factor in a clinical study performed at our institution [25].

Our findings suggest that only the use of a panel of markers will allow the consistent detection of microscopic LN metastases, since not all markers are necessarily coexpressed. This might also have implications for the development of liquid biopsy panels. The most common liquid biopsy biomarkers are CTCs, circulating tumor DNA (ctDNA), and extracellular vesicles. It was recently shown that the levels of ctDNA are below the threshold for detection in serially collected plasma samples over 24 mo after RP and therefore not suitable for the detection of recurrence [26], while it very well recapitulates the genomic landscape detected in tissue biopsies of metastatic PC [27]. The number of CTCs positively correlates with biochemical recurrence after RP; yet, there is considerable phenotypic heterogeneity regarding the expression of androgen receptor and cytokeratine [28]. It is possible that a more extensive panel of prostate epithelial markers could improve the utility of CTCs as predictors of recurrence.

Our hypothesis that markers of stemness and EMT may be more sensitive than epithelial markers in detecting micrometastasis could not be validated, since all the markers analyzed were already expressed in control LNs to the same or an even higher extent than in metastatic LNs. Our choice of SC markers was based on our report in the BM18 xenograft model of PC that cells surviving castration and able to reinitiate tumor growth are characterized by the coexpression of ALDH1A1 or NANOG together with the luminal marker NKX3-1 [16]. These castration-resistant cells also show mRNA expression of OCT4 and SOX2, LGR5 and LGR6, KLF4, EGR1, BMI1, LRIG1, and TSPAN7 and TSPAN13. For the markers of EMT used, there is a large body of evidence that SNAIL, TWIST, and CXCR4 are representative of PC, and correlate with metastases and poor outcome [29], [30]. The presence of EMT, as assessed by the coexpression of CK8 and vimentin, was predictive of biochemical recurrence after RP, irrespective of other clinical factors such as Gleason grade, pathological stage, or surgical margins [31].

The concept of the tumor microenvironment as a decisive factor in the metastatic process is well established. However, similarly to what was observed with markers for stemness and EMT, the markers of reactive stroma did not discriminate between metastatic and control LNs. Our analysis was limited to four genes, *ASPN*, *POSTN*, *SPARCL1*, and *MCAM*, which we determined as potential markers of stromal activation in a xenograft model of PC bone metastasis [17]. It is conceivable that other stroma markers might be more suitable for the detection of LN metastases.

Potential limitations of our work are the cutting of the LN in half, which might miss tumor foci in the histopathological or molecular workup as well as the hypothesis-driven selection of markers that might differ from markers selected by high throughput screening.

## 5 Conclusions

In conclusion, the expression of markers of stemness, EMT, and reactive stroma in the LN microenvironment precludes their use in the detection of micrometastases. Consequently, prostate epithelial markers remain the best candidates for RP with LN dissection. Additional trials are needed to validate the clinical utility of molecular detection of epithelial prostate markers as prognostic biomarkers.

  ***Author contributions*:** Bernhard Kiss had full access to all the data in the study and takes responsibility for the integrity of the data and the accuracy of the data analysis.

*Study concept and design*: Thalmann.

*Acquisition of data*: Özdemir, Arnold, Hensel, Klima.

*Analysis and interpretation of data*: Özdemir, Arnold, Kiss, Fleischmann.

*Drafting of the manuscript*: Özdemir, Arnold, Kiss, Burkhard, Kruithof-deJulio, Thalmann.

*Critical revision of the manuscript for important intellectual content*: Özdemir, Arnold, Kiss, Thalmann.

*Statistical analysis*: Hayoz.

*Obtaining funding*: None.

*Administrative, technical, or material support*: None.

*Supervision*: Kiss, Thalmann.

*Other*: None.

  ***Financial disclosures:*** Bernhard Kiss certifies that all conflicts of interest, including specific financial interests and relationships and affiliations relevant to the subject matter or materials discussed in the manuscript (eg, employment/affiliation, grants or funding, consultancies, honoraria, stock ownership or options, expert testimony, royalties, or patents filed, received, or pending), are the following: None.

  ***Funding/Support and role of the sponsor*:** This study was supported by a grant from the Swiss National Foundation (320030_130862).

  ***Acknowledgments*:** The authors are grateful to Nathalie Tschan for retrieval of clinical follow-up data and to the members of the Institute of Pathology for collecting the lymph nodes. They also thank the Department of Clinical Research, University of Bern, Switzerland, for logistic support.

## References

[1] van der Toom E.E., Verdone J.E., Pienta K.J. (2016). Disseminated tumor cells and dormancy in prostate cancer metastasis. Curr Opin Biotechnol.

[2] Fleischmann A., Schobinger S., Schumacher M., Thalmann G.N., Studer U.E. (2009). Survival in surgically treated, nodal positive prostate cancer patients is predicted by histopathological characteristics of the primary tumor and its lymph node metastases. Prostate.

[3] Dorin R.P., Lieskovsky G., Fairey A.S., Cai J., Daneshmand S. (2013). Outcomes after radical prostatectomy for patients with clinical stages T1–T2 prostate cancer with pathologically positive lymph nodes in the prostate-specific antigen era. Urol Oncol.

[4] Clobes H., Fossa S.D., Waehre H., Jocham D., Berner A. (2000). The immunohistochemical assessment of occult regional lymph node metastases in patients with T3pN0M0 prostate cancer before definitive radiotherapy. BJU Int.

[5] Shariat S.F., Roudier M.P., Wilcox G.E. (2003). Comparison of immunohistochemistry with reverse transcription-PCR for the detection of micrometastatic prostate cancer in lymph nodes. Cancer Res.

[6] Okegawa T., Nutahara K., Higashihara E. (2000). Detection of micrometastatic prostate cancer cells in the lymph nodes by reverse transcriptase polymerase chain reaction is predictive of biochemical recurrence in pathological stage T2 prostate cancer. J Urol.

[7] Terakawa T., Miyake H., Kurahashi T., Furukawa J., Takenaka A., Fujisawa M. (2009). Improved sensitivity for detecting micrometastases in pelvic lymph nodes by real-time reverse transcriptase polymerase chain reaction (RT-PCR) compared with conventional RT-PCR in patients with clinically localized prostate cancer undergoing radical prostatectomy. BJU Int.

[8] Adsan O., Cecchini M.G., Bisoffi M. (2002). Can the reverse transcriptase-polymerase chain reaction for prostate specific antigen and prostate specific membrane antigen improve staging and predict biochemical recurrence?. BJU Int.

[9] Heck M.M., Retz M., Bandur M. (2018). Molecular lymph node status for prognostic stratification of prostate cancer patients undergoing radical prostatectomy with extended pelvic lymph node dissection. Clin Cancer Res.

[10] Pagliarulo V., Hawes D., Brands F.H. (2006). Detection of occult lymph node metastases in locally advanced node-negative prostate cancer. J Clin Oncol.

[11] Satelli A., Batth I., Brownlee Z. (2017). EMT circulating tumor cells detected by cell-surface vimentin are associated with prostate cancer progression. Oncotarget.

[12] Shiozawa Y., Berry J.E., Eber M.R. (2016). The marrow niche controls the cancer stem cell phenotype of disseminated prostate cancer. Oncotarget.

[13] Harner-Foreman N., Vadakekolathu J., Laversin S.A. (2017). A novel spontaneous model of epithelial-mesenchymal transition (EMT) using a primary prostate cancer derived cell line demonstrating distinct stem-like characteristics. Sci Rep.

[14] Klarmann G.J., Hurt E.M., Mathews L.A. (2009). Invasive prostate cancer cells are tumor initiating cells that have a stem cell-like genomic signature. Clin Exp Metastasis.

[15] Ayala G., Tuxhorn J.A., Wheeler T.M. (2003). Reactive stroma as a predictor of biochemical-free recurrence in prostate cancer. Clin Cancer Res.

[16] Germann M., Wetterwald A., Guzman-Ramirez N. (2012). Stem-like cells with luminal progenitor phenotype survive castration in human prostate cancer. Stem Cells.

[17] Ozdemir B.C., Hensel J., Secondini C. (2014). The molecular signature of the stroma response in prostate cancer-induced osteoblastic bone metastasis highlights expansion of hematopoietic and prostate epithelial stem cell niches. PLoS One.

[18] Ruppender N.S., Morrissey C., Lange P.H., Vessella R.L. (2013). Dormancy in solid tumors: implications for prostate cancer. Cancer Metastasis Rev.

[19] Guzman-Ramirez N., Voller M., Wetterwald A. (2009). In vitro propagation and characterization of neoplastic stem/progenitor-like cells from human prostate cancer tissue. Prostate.

[20] Xiang Q., Zhu Z., Luo L. (2020). The correlation between PSCA expression and neuroendocrine differentiation in prostate cancer. Biomed Res Int.

[21] Dorff T.B., Blanchard S., Carruth P. (2020). A phase I study to evaluate PSCA-targeting chimeric antigen receptor (CAR)-T cells for patients with PSCA+ metastatic castration-resistant prostate cancer (mCRPC). J Clin Oncol.

[22] Hu Z., Gu Y., Han B. (2012). Knockdown of AGR2 induces cellular senescence in prostate cancer cells. Carcinogenesis.

[23] Gurel B., Ali T.Z., Montgomery E.A. (2010). NKX3.1 as a marker of prostatic origin in metastatic tumors. Am J Surg Pathol.

[24] Hsu E.C., Rice M.A., Bermudez A. (2020). Trop2 is a driver of metastatic prostate cancer with neuroendocrine phenotype via PARP1. Proc Natl Acad Sci U S A.

[25] Fleischmann A., Saramaki O.R., Zlobec I. (2014). Prevalence and prognostic significance of TMPRSS2-ERG gene fusion in lymph node positive prostate cancers. Prostate.

[26] Hennigan S.T., Trostel S.Y., Terrigino N.T. (2019). Low abundance of circulating tumor DNA in localized prostate cancer. JCO Precis Oncol.

[27] Tukachinsky H., Madison R.W., Chung J.H. (2021). Genomic analysis of circulating tumor DNA in 3,334 patients with advanced prostate cancer identifies targetable BRCA alterations and AR resistance mechanisms. Clin Cancer Res.

[28] Salami S.S., Singhal U., Spratt D.E. (2019). Circulating tumor cells as a predictor of treatment response in clinically localized prostate cancer. JCO Precis Oncol.

[29] Borretzen A., Gravdal K., Haukaas S.A. (2021). The epithelial-mesenchymal transition regulators TWIST, SLUG, and SNAIL are associated with aggressive tumour features and poor outcome in prostate cancer patients. J Pathol Clin Res.

[30] Chen Q., Zhong T. (2015). The association of CXCR4 expression with clinicopathological significance and potential drug target in prostate cancer: a meta-analysis and literature review. Drug Des Devel Ther.

[31] Cheaito K.A., Bahmad H.F., Hadadeh O. (2019). EMT markers in locally-advanced prostate cancer: predicting recurrence?. Front Oncol.

