# Supplementary material for: Prediction of Biochemical Recurrence Based on Molecular Detection of Lymph Node Metastasis After Radical Prostatectomy
Source: Eur Urol Open Sci. 2022 Aug 16;44:1–10. doi: 10.1016/j.euros.2022.07.005 (PMC9520506; doi:10.1016/j.euros.2022.07.005)
Supplement: Supplementary data 1 [file mmc1.docx]

**Supplementary Table 1 *Gene symbols and expression assays***

| \| Gene ^a)^ \| Abbreviation \| Assay ^b)^ \| \|  \| \| \| --- \| --- \| --- \| --- \| --- \| --- \| \| β-actin \| *ACTB* \| Hs99999903_m1 \|  \| \| \| Anterior gradient 2 homolog \| *AGR2* \| Hs00180702_m1 \|  \| \| \| Aldehyde dehydrogenase 1A1 \| *ALDH1A1* \| Hs00946916_m1 \|  \| \| \| Asporin \| *ASPN* \| Hs00214395_m1 \|  \| \| \| Polycomb complex protein BMI-1 \| *BMI1* \| Hs00180411_m1 \|  \| \| \| Chemokine (C-X-C motif) receptor 4 \| *CXCR4* \| Hs00237052_m1 \|  \| \| \| Early growth response 1 \| *EGR1* \| Hs00152928_m1 \|  \| \| \| Epithelial cell adhesion molecule \| *EpCAM* \| Hs00158980_m1 \|  \| \| \| Glyceraldehyde-3-phosphate dehydrogenase \| *GAPDH* \| Hs99999905_m1 \|  \| \| \| Kruppel-like factor 4 \| *KLF4* \| Hs00358836_m1 \|  \| \| \| Leucine-rich repeat containing G protein-coupled receptor 5 \| *LGR5* \| Hs00173664_m1 \|  \| \| \| Leucine-rich repeat containing G protein-coupled receptor 6 \| *LGR6* \| Hs00663887_m1 \|  \| \| \| Leucine-rich repeats and immunoglobulin-like domains 1 \| *LRIG1* \| Hs00394267_m1 \|  \| \| \| Melanoma cell adhesion molecule \| *MCAM* \| Hs00174838_m1 \|  \| \| \| Homeobox protein NANOG \| *NANOG* \| Hs02387400_g1 \|  \| \| \| Homeobox protein Nkx-3.1 \| *NKX3-1* \| Hs00171834_m1 \|  \| \| \| Octamer-binding protein 4A \| *OCT4* \| Hs03005111_g1 \|  \| \| \| Periostin, osteoblast specific factor \| *POSTN* \| Hs00170815_m1 \|  \| \| \| Prostate specific antigen \| *PSA* \| Hs00426859_g1 \|  \| \| \| Prostate stem cell antigen \| PSCA \| Hs00194665_m1 \|  \| \| \| Prostate-specific membrane antigen \| PSMA \| Hs00194665_m1 \|  \| \| \| Snail homolog 1 \| SNAIL \| Hs00195591_m1 \|  \| \| \| Transcription factor SOX-2 \| SOX2 \| Hs01053049_s1 \|  \| \| \| SPARC-like 1 (hevin) \| SPARCL1 \| Hs00190740_m1 \|  \| \| \| Tumor-associated calcium signal transducer 2 \| TROP2 \| Hs01922976_s1 \|  \| \| \| Tetraspanin 7 \| TSPAN7 \| Hs00190284_m1 \|  \| \| \| Tetraspanin 13 \| TSPAN13 \| Hs00917717_m1 \|  \| \| \| TMPRSS2-ERG prostate cancer specific \| TMPRSS-ERG \| Hs03063375_ft \|  \| \| \| Twist homolog 1 \| TWIST \| Hs01675818_s1 \|  \| \| \| ^a)^ Gene names according to HUGO Gene Nomenclature Committee (<http://www.genenames.org/>); ^b)^ Applied Biosystems \| \| \| \| \| |  |  |
| --- | --- | --- | --- | --- | --- | --- | --- | --- | --- | --- | --- | --- | --- | --- | --- | --- | --- | --- | --- | --- | --- | --- | --- | --- | --- | --- | --- | --- | --- | --- | --- | --- | --- | --- | --- | --- | --- | --- | --- | --- | --- | --- | --- | --- | --- | --- | --- | --- | --- | --- | --- | --- | --- | --- | --- | --- | --- | --- | --- | --- | --- | --- | --- | --- | --- | --- | --- | --- | --- | --- | --- | --- | --- | --- | --- | --- | --- | --- | --- | --- | --- | --- | --- | --- | --- | --- | --- | --- | --- | --- | --- | --- | --- | --- | --- | --- | --- | --- | --- | --- | --- | --- | --- | --- | --- | --- | --- | --- | --- | --- | --- | --- | --- | --- | --- | --- | --- | --- | --- | --- | --- | --- | --- | --- | --- | --- | --- | --- | --- | --- | --- | --- | --- | --- | --- | --- | --- | --- | --- | --- | --- | --- | --- | --- | --- | --- | --- | --- | --- | --- | --- | --- | --- | --- | --- | --- | --- | --- |

**Supplementary Table 2 *Primary antibodies used for immunohistochemistry***

| Antibodies | Company | Catalog number | Clone | Concentration ^a)^ | Antigen retrieval |
| --- | --- | --- | --- | --- | --- |
| AGR2 | Epitomics | 2574 | EPR3278 | 1:500 | Citrate |
| EpCAM | DAKO | M3525 | MOC-31 | 1:100 | Citrate |
| NKX3-1 | AthenaES | 314 |  | 1μg/ml | EDTA |
| PSA | DAKO | A0562 |  | 1μg/ml | Citrate |
| PSCA | Invitrogen | 18-7371 |  | 3μg/ml | EDTA |
| PSMA | Invitrogen | 34-4100 |  | 0.5μg/ml | EDTA |
| ^a)^ Working concentrations are indicated where known; dilutions are given where the antibody concentration is not specified by the manufacturer | | | | | |

**Supplementary Table 3 *Gene expression in control patients***

|  | | mean | SD | mean + 2SD |
| --- | --- | --- | --- | --- |
| Prostate epithelial markers |  |  |  |  |
| *PSA* |  | 3.36 | 6.43 | 16.23 |
| *PSCA* |  | 0.42 | 0.79 | 1.99 |
| *EpCAM* |  | 0.16 | 0.20 | 0.57 |
| *PSMA* |  | 0.50 | 0.44 | 1.39 |
| *NKX3-1* |  | 2.72 | 3.27 | 9.27 |
| *AGR2* |  | 0.00 | 0.00 | 0.00 |
| Stem cell markers | |  | |  |
| *ALDH1A1* |  | 112.75 | 93.74 | 300.23 |
| *BMI1* |  | 22.50 | 7,36 | 37.21 |
| *KLF4* |  | 421.89 | 582.74 | 1587.38 |
| *LGR5* |  | 0.22 | 0.19 | 0.60 |
| *LGR6* |  | 1.58 | 1.03 | 3.63 |
| *LRIG1* |  | 84.22 | 78.63 | 241.48 |
| *NANOG* |  | 269.88 | 603.96 | 1477.80 |
| *OCT4* |  | 5.40 | 1.47 | 8.35 |
| *SOX2* |  | 0.29 | 0.17 | 0.65 |
| *TROP2* |  | 0.12 | 0.19 | 0.50 |
| *TSPAN13* |  | 118.86 | 65.94 | 250.74 |
| *TSPAN7* |  | 75.12 | 34.06 | 143.25 |
| EMT markers | |  |  |  |
| *CXCR4* |  | 68.02 | 66.44 | 200.89 |
| *SNAIL* |  | 4.90 | 5.56 | 16.0 |
| *TWIST* |  | 20.67 | 20.38 | 61.42 |
| Reactive stroma markers | |  |  |  |
| *ASPN* |  | 8.88 | 7.53 | 23.94 |
| *MCAM* |  | 133.47 | 116.48 | 366.44 |
| *POSTN* |  | 71.90 | 96.06 | 264.01 |
| *SPARCL1* |  | 471.50 | 231.51 | 934.53 |
| n=5 control patients, 11 lymph nodes | | | | |

**Supplementary Table 4. *Gene expression in lymph nodes from patients with PSA relapse***

| Patient | *PSA*^pos^ | *PSCA*^pos^ | *EpCAM*^pos^ | *PSMA*^pos^ | *NKX3-1*^pos^ |
| --- | --- | --- | --- | --- | --- |
| pN0 |  |  |  |  |  |
| 144 | 0/12 | 0/12 | 0/12 | 0/12 | 0/12 |
| 162 | 0/9 | 0/9 | 0/9 | 0/12 | 0/9 |
| 174 | 7/13 | 0/13 | 7/13 | 10/13 | 6/13 |
| 178 | 0/27 | 0/27 | 0/27 | 9/27 | 4/27 |
| 210 | 0/18 | 0/18 | 0/18 | 0/18 | 1/18 |
| 246 | 0/20 | 0/20 | 0/20 | 0/20 | 0/20 |
| pN1 |  |  |  |  |  |
| 130 | 11/13 | 0/13 | 11/13 | 12/13 | 10/13 |
| 135 | 0/25 | 0/25 | 1/25 | 5/25 | 0/25 |
| 138 | 2/10 | 2/10 | 6/10 | 5/8 | 2/8 |
| 147 | 6/15 | 3/15 | 4/15 | 8/15 | 4/15 |
| 194 | 7/19 | 7/19 | 4/19 | 12/19 | 7/19 |
| 204 | 3/20 | 0/20 | 3/20 | 1/20 | 2/20 |
| 227 | 12/17 | 12/17 | 13/17 | 14/17 | 12/17 |
| 229 | 3/18 | 3/18 | 3/18 | 3/18 | 3/18 |
| 240 | 8/15 | 6/15 | 8/15 | 10/15 | 8/15 |
| PSA = prostate-specific antigen; EpCAM = epithelial cell adhesion molecule; PSCA = prostate stem cell antigen; PSMA = prostate-specific membrane antigen; NKX3-1 = homeobox protein Nkx-3.1 | | | | | |
